# Toxicity of *Ocimum basilicum* and *Ocimum gratissimum* Extracts against Main Malaria Vector, *Anopheles gambiae* (Diptera: Culicidae) in Nigeria

**Published:** 2019-12-31

**Authors:** Kayode David Ileke, Jacobs Mobolade Adesina

**Affiliations:** 1 Department of Biology, School of Science, Federal University of Technology, Akure, Ondo State, Nigeria; 2 Department of Crop, Soil and Pest Management Technology, Rufus Giwa Polytechnic, Owo, Ondo State, Nigeria; 3 Insect Chemical Ecology Laboratory, Institute of Bioresources and Sustainable Development, Department of Biotechnology, Govt. of India, Takyelpat, Manipur, India

**Keywords:** *Ocimum basilicum*, *Ocimum gratissimum*, Insecticide, *Anopheles gambiae*, *Clarias garipienus*

## Abstract

**Background:**

*Anopheles gambiae* (Diptera: Culicidae) transmit malaria parasite that causes malaria fever in humans, causing millions of deaths every year among infants in tropical countries. This study was undertaken to assess the toxicity of *Ocimum basilicum* and *Ocimum gratissimum* against pre-adult stages and adult malaria vector, *An. gambiae* and non-targeted aquatic organism, fingerlings of *Clarias garipienus*.

**Methods:**

Ethalonic extracts of *O. basilicum* and *O. gratissimum* were prepared according to the method described by WHO. The larvae and pupae of *An. gambiae* were exposed to plant extracts for 24h and their mortality was recorded. Toxicity of *Ocimum* species on non-targeted organism, fingerlings of *C. garipienus* was also investigated.

**Results:**

*Ocimum basilicum* showed remarkably potency against pre-adult stages and adults *An. gambiae* causing 100% mortality at 0.4% concentration within 24h of treatment. The LC_50_ and LC_90_ of *O. basilicum* were lower than *O. grattisimum* in all stages of *An. gambiae* studied. *Ocimum basilicum* and *O. gratissimum* extracts significantly reduced the number of bites by the vector given a range of 72.25% to 81.75% protection. *Ocimum* species at the tested concentrations did not significantly reduce the number of fingerlings introduced.

**Conclusion:**

*Ocimum* species at the tested concentrations did not significantly reduce the numbers of non-targeted organisms, fingerlings introduced. Therefore, *O. basilicum* and *O. gratissimum* could be used to reduce malaria prevalence in the endemic areas of Nigeria as it poses no threat to aquatic organisms.

## Introduction

Arthropod insect vectors are blamable for spreading serious human diseases like malaria, encephalitis, yellow fever, dengue and filariasis (1). In Africa, malaria is a vector-borne infectious disease that causes the death of infants and this had led to public health concern throughout the tropical and subtropical regions (2). Alteration in the natural environment have also contributed to the widespread of the disease and change in behavior of the vector toward chemical insecticides.

In the beginning, synthetic chemical insecticide proves to be the only effective means of combating mosquito until its adverse effects on the environment and user health become pronounce (3). Malaria vectors have developed a resistant mechanism against chemical insecticides because of continuous application of synthetic products in our environment for their management (1, 4, 5). The problems of these environmental and health hazards limit their success in vector control, which has led to the development of eco-friendly, biodegradable and readily available plant-based mosquitocides with low-cost implication (3, 6, 7). Botanicals are considered as one of the harmless sources for controlling insect vectors and stored products pests (8, 9). Botanical based insecticides have been reported by many entomologists and parasitologists to control larvae, pupae, and adult mosquitoes (8, 10, 11).

*Ocimum* species belong to the family Lamiaceae. Recent research has investigated the health benefits associated with *Ocimum* essential oils. Studies revealed the anti-viral, anti-microbial, antioxidant, and anti-cancer properties of the plants (12).

Hence, in view of an increasing interest in developing plant-based insecticides as an alternative to chemical mosquitocides, this study was undertaken to assess the toxicity of *O. basilicum* and *O. gratissimum* against pre-adult stages and adult malaria vector, *Anopheles gambiae* and non-targeted aquatic organism, fingerlings of *Clarias garipienus*.

## Materials and Methods

### Mosquito rearing

*Anopheles gambiae* mosquitoes were breed in the Hatchery Laboratory, Department of Animal and Environmental Biology, Adekunle Ajasin University Akungba Akoko, Ondo State, Nigeria (7° 28′ N, 5° 44′ E) and maintained at ambient temperature of 28±2 °C with 12:12 light and dark photoperiod in 75±5% relative humidity.

### Plant Materials and Extractions

*Ocimum gratissimum* and *O. basilicum* leaves were collected fresh from Supare Akoko, Ondo State (7° 26′ 0″ North, 5° 43′ E). Plant leaves were authenticated by Plant Taxonomist in the Plant Science and Biotechnology Department, Adekunle Ajasin University, Akungba Akoko, Ondo State (7° 28′ N, 5° 44′ E). The leaves were rinsed in distilled water to remove any form of impurities, air-dried in laboratory and ground into powder.

About 150g of *O. gratissimum* and *O. basilicum* leaves powders were soaked separately in an extraction bottle containing absolute ethanol. Stirred occasionally with a glass rod and extraction terminated after 3 days. The resulting mixture was filtered and the solvent was evaporated using a rotary evaporator. Extracts were kept in a vial and preserved in the refrigerator until further use.

### Larval, Pupal and Adult Mortality Bioassay

Bioassay tests were carried out on *Ocimum* species extracts using five different concentrations (0.1%, 0.2%, 0.3%, 0.4% and 0.5%) prepared according to the standard methods recommended by WHO (13–15). Twenty larvae and pupae of *An. gambiae* were separately introduced into the treated water, solvent treated and untreated water was set as control. Each treatment was replicated four times. Mortality was observed over 24h of treatment.

Twenty *An. gambiae* adults were introduced into a test-tube that contain suspended filter papers soaked with 0.1%, 0.2%, 0.3%, 0.4% and 0.5% *Ocimum* species extracts separately in four replicates for adult bioassay according (2, 3). Mortality of adult insect was accessed after 2h of post-exposure. Percentage larvae, pupae and adult mortality were corrected (16).

### Mosquito Coil Toxicity Bioassay

Chemical mosquito coils were mimic to form plant-based coils derived from *O. basilicum* and *O. gratissimum* separately. This is done by thoroughly mixed 10ml of 50% concentrated plant materials with 5g coconut shell, charcoal powder and distilled water to form semi-solid material that solidify with time under shade (3). Coil toxicity bioassay was evaluated using a glass chamber size 100× 70× 30cm. Two control coils were also set up, one made without extracts and the second control made using synthetic chemical as positive control. One hundred 2–3d old adult *An. gambiae*, fed with 10% sucrose solution was released into the chamber for 60min. The procedure was repeated four times on separate days. Mortality of adult insect was accessed after 60min of post-exposure. Percentage adult mortality was corrected using the methods described by Abbott (16).

### Non-targeted Aquatic organism Bioassay

Fingerlings of *C. garipienus* of not more than 4–5wk old were collected from the Hatchery Laboratory, Department of Animal and Environmental Biology, Adekunle Ajasin University Akungba Akoko, Ondo State, Nigeria (7° 28′ N, 5° 44′ E). The fingerlings were acclimatized for 14d in a dechlorinated borehole water. Fingerlings were fed twice daily until 24h prior to their exposure to *Ocimum* species in accordance with the recommendation of Adekunle Ajasin University, Akungba Akoko Ondo State, Nigeria Ethical Committee*.* Twenty fingerlings were exposed to each concentration tested, fed with fish pellets (1mm) and daily monitored for 5d. Mortality was recorded daily for four days. They were considered dead when no visible movement was observed when agitated and probe with a sharp object.

### Analysis of Data

The percentage of mortality of larvae, pupae and adults were calculated and corrected relative to the associated controls using Abbott’s formula. Lethal concentrations (LC_50_ and LC_90_) and their 95% confidence limits were determined using Probit analysis (17).

## Result

### Larvicidal, Pupicidal and Adulticidal activity of *Ocimum grattisimum* and *O. basilicum* extracts

*Ocimum grattisimum* and *O. basilicum* significantly affects the mortality of *An. gambiae* larvae at all the concentrations tested (Table 1). The toxicity of *O. grattisimum* and *O. basilicum* leaves extracts were significantly (P< 0.05) different from the solvent treated and control experiment. *Ocimum basilicum* extract was the most toxic causing 100% mortality at 0.4% concentration within 24h of post-exposure and its effect was not significantly (P< 0.05) different from *O. grattisimum* extract. *Ocimum grattisimum* and *O. basilicum* extracts were able to cause 100% mortality at 0.5% concentration within 24h of post exposure and its effect was significantly (P< 0.05) different from solvent treated and control.

**Table 1.** Toxicity of *Ocimum grattisimum* and *O. basilicum* on Larvae of *Anopheles gambiae*

**Plant Extracts**	**Concentration (%)**				

	**0.1**	**0.2**	**0.3**	**0.4**	**0.5**
***Ocimum grattisimum***	55.00±3.75^b^	65.00±3.75^b^	85.00±3.75^b^	90.00±4.25^b^	100.00±0.00^b^
***Ocimum basilicum***	60.00±4.25^b^	75.00±3.75^b^	92.50±4.20^b^	100.00±0.00^b^	100.00±0.00^b^
**Solvent treated**	0.00±0.00^a^	0.00±0.00^a^	0.00±0.00^a^	0.00±0.00^a^	0.00±0.00^a^
**Untreated**	0.00±0.00^a^	0.00±0.00^a^	0.00±0.00^a^	0.00±0.00^a^	0.00±0.00^a^

Each value is a mean ± standard error of four replicates. Means followed by the same letter along the column are not significantly different using Duncan’s new multiple range test.

Similarly, *O. basilicum* caused 100% pupae mortality within 24h of treatment at 0.5% concentration and its effect was significantly not different from *O. grattisimum* extract who caused 90% pupae mortality (Table 2).

**Table 2.** Toxicity of *Ocimum grattisimum* and *O. basilicum* on Pupae of *Anopheles gambiae*

**Plant Extracts**	**Concentration (%)**				

	**0.1**	**0.2**	**0.3**	**0.4**	**0.5**
***Ocimum grattisimum***	45.00±3.75^b^	50.00±4.25^b^	70.00±4.25^b^	77.50±4.10^b^	90.00±4..25^b^
***Ocimum basilicum***	50.00±4.25^b^	60.00±4.25^b^	75.00±3.75^b^	82.50±4.20^b^	100.00±0.00^b^
**Solvent treated**	0.00±0.00^a^	0.00±0.00^a^	0.00±0.00^a^	0.00±0.00^a^	0.00±0.00^a^
**Untreated**	0.00±0.00^a^	0.00±0.00^a^	0.00±0.00^a^	0.00±0.00^a^	0.00±0.00^a^

Each value is a mean ± standard error of four replicates. Means followed by the same letter along the column are not significantly different using Duncan’s new multiple range test.

The results of fumigant toxicity of *O. basilicum* leaf extract were able to achieved 100% insect mortality at 0.5% concentration while *O. grattisimum* caused 95% adult mortality after 60 min of exposure period (Table 3). *Ocimum grattisimum* and *O. basilicum* leaves extracts effects were significantly (P< 0.05) different from solvent treated and the control at all tested concentrations.

**Table 3.** Fumigant Toxicity of *Ocimum grattisimum* and *O. basilicum* on *Anopheles gambiae* Adults

**Plant Extracts**	**Concentration (%)**				

	**0.1**	**0.2**	**0.3**	**0.4**	**0.5**
***Ocimum grattisimum***	50.00±4.25^b^	60.00±4.25^b^	75.00±3.75^b^	85.00±4.25^b^	95.00±3.75^c^
***Ocimum basilicum***	60.00±4.25^b^	75.00±3.75^b^	85.00±4.20^b^	95.00±0.00^b^	100.00±0.00^c^
**Solvent treated**	0.00±0.00^a^	0.00±0.00^a^	0.00±0.00^a^	0.00±0.00^a^	10.00±0.25^b^
**Untreated**	0.00±0.00^a^	0.00±0.00^a^	0.00±0.00^a^	0.00±0.00^a^	0.00±0.00^a^

Each value is a mean ± standard error of four replicates. Means followed by the same letter along the column are not significantly different using New Duncan’s multiple Range test

The LC_50_ and LC_90_ of *O. basilicum* were lower than *O. grattisimum* in all stages of *An. gambiae* studied (Table 4). The lethal concentration of *O. grattisimum* and *O. basilicum* to achieve 50% mortality was lower in larvae stage (0.104% and 0.093% respectively) compared to pupae (0.145% and 0.121% respectively) and adult (0.118% and 0.088% respectively) of *An. gambiae*. Similarly, the lethal concentration of *O. grattisimum* and *O. basilicum* to achieve 9 % mortality was lower in larvae stage (0.363% and 0.246% respectively) compared to pupae (0.728 % and 0.485% respectively) and adult (0.519 % and 0.305% respectively) of *An. gambiae*.

**Table 4.** LC_50_ and LC_90_ of *Ocimum grattisimum* and *O. basilicum* extracts

***An. gambiae***	**Plants**	**LC_50_ (LCL–UCL)%**	**LC_90_ (LCL–UCL)%**
**Larvae**	*O. grattisimum*	0.104 (0.007–0.168)	0.363 (0.232–0.494)
	*O. basilicum*	0.093 (0.013–0.143)	0.246 (0.165–0.770)
**Pupae**	*O. grattisimum*	0.145 (0.025–0.223)	0.728 (0.409–1.047)
	*O. basilicum*	0.121 (0.032–0.210)	0.485 (0.273–0.697)
**Adults**	*O. grattisimum*	0.118 (0.026–0.180)	0.519 (0.330–0.708)
	*O. basilicum*	0.088 (0.013–0.140)	0.305 (0.208–0.904)

Keys: LC Lethal Concentration, LCL–Lower Concentration Limit, UCL–Upper Concentration Limit

### Mosquito Coil Toxicity

Smoke toxicity of *O. grattisimum* and *O. basilicum* extracts on *An. gambiae* adults were presented in Table 5. *Ocimum grattisimum* and *O. basilicum* extracts and the positive control (Baygon insecticide) were not able to give 100% protection against adult *A. gambiae* from sucking blood. Positive control (Baygon insecticide) had the lowest number of fed mosquito (10.5). This is followed by *O. basilicum* extract which had 6.75 and 11.5 fed mosquitoes in *O. grattisimum* extract which was not significantly different from other treatments apart from untreated control that recorded 47.5 fed *An. gambiae*. *Ocimum grattisimum*, *O. basilicum* extracts and positive control were able to achieve 72.25%, 81.75% and 84.5% Protection which was not significantly from each other.

**Table 5.** Smoke toxicity of *Ocimum grattisimum* and *O. basilicum* extracts on Adults *An. Gambiae*

**Extracts**	**Total number of Adult mosquitoes**	**Fed Mosquitoes**	**Unfed Mosquitoes**	**% Protection**
***Ocimum grattisimum***	50	11.50±1.15^a^	38.50±2.24^b^	72.25±3.24^b^
***Ocimum basilicum***	50	6.75±0.08^a^	43.25±2.44^b^	81.75±3.18^b^
**Control I (Synthetic Insecticide)**	50	5.25±0.35^a^	44.75±2.37^b^	84.50±3.39^b^
**Control II (Untreated)**	50	47.50±2.20^b^	2.50±0.04^a^	0.00 ± 0.00^a^

Each value is a mean ± standard error of four replicates. Means followed by the same letter along the column are not significantly different using New Duncan’s multiple Range test

### Toxicity of *Ocimum basilicum* and *O. gratis-simum* Extracts on Aquatic Habitat organism

There was no mortality of fingerlings recorded after 2d of water treatment (Table 6). However, 10.25% mortality were recorded on fingerlings water treated with 0.5% *O. grattisimum* and *O. basilicum* extracts after 3d of treatment. Similarly, 10% and mortality of finger-lings were recorded in water treated with 0.4 % *O. grattisimum* and *O. basilicum* extracts after 4d of exposure period. At 0.5% concentration, 20% mortality were recorded in fingerlings water treated with *O. grattisimum* and *O. basilicum* extracts after 5 d of treatment.

**Table 6.** Toxicity of Fingerlings treated with *Ocimum grattisimum* and *O. basilicum* extracts

**Extracts of plants Conc.**	**Mortality%±S. E after**				

	**1day**	**2 days**	**3 days**	**4 days**	**5 days**
***Ocimum grattisimum***					
**0.1**	0.00±0.00^a^	0.00±0.00^a^	0.00±0.00^a^	0.00±0.00^a^	0.00±0.00^a^
**0.2**	0.00±0.00^a^	0.00±0.00^a^	0.00±0.00^a^	0.00±0.00^a^	0.00±0.00^a^
**0.3**	0.00±0.00^a^	0.00±0.00^a^	0.00±0.00^a^	0.00±0.00^a^	10.00±0.25^b^
**0.4**	0.00±0.00^a^	0.00±0.00^a^	0.00±0.00^a^	10.00±0.04^b^	10.00±0.04^b^
**0.5**	0.00±0.00^a^	0.00±0.00^a^	10.00±0.04^b^	10.00±0.04^b^	20.00±1.14^b^
***Ocimum basilicum***					
**0.1**	0.00±0.00^a^	0.00±0.00^a^	0.00±0.00^a^	0.00±0.00^a^	0.00±0.00^a^
**0.2**	0.00±0.00^a^	0.00±0.00^a^	0.00±0.00^a^	0.00±0.00^a^	0.00±0.00^a^
**0.3**	0.00±0.00^a^	0.00±0.00^a^	0.00±0.00^a^	0.00±0.00^a^	10.00±0.25^b^
**0.4**	0.00±0.00^a^	0.00±0.00^a^	0.00±0.00^a^	10.00±0.25^b^	10.00±0.25^b^
**0.5**	0.00±0.00^a^	0.00±0.00^a^	10.25±0.04^b^	10.00±0.02^b^	20.00±1.14^b^
**Untreated**	0.00±0.00^a^	0.00±0.00^a^	0.00±0.00^a^	0.00±0.00^a^	0.00±0.00^a^

Each value is a mean ± standard error of four replicates. Means followed by the same letter along the column are not significantly different using Duncan’s new multiple range test

## Discussion

The use of plant-derived products as mosquitocides have proved to be an alternative approach to the control of insect vectors since the use of synthetic insecticides have been discouraged due to their food and environmental health concerns, toxicity to untargeted organisms and insect vector resurgence rates have made their exploitation undesirable (18).

According to the results of our study, the extracts of the *Ocimum* species caused high mortality of *An. gambiae* larvae, pupae and adults. The high lethal effects on pre-adults and adults *An. gambiae* may be ascribed to the active compounds presents in these plants such as alkaloids (8). The LC_50_ and LC_90_ of *O. basilicum* were lower than *O. grattisimum* in all stages of *An. gambiae* studied. The lethal concentration of *O. grattisimum* and *O. basilicum* to achieve 50% mortality was lower in larvae stage (0.104% and 0.093% respectively) compared to pupae (0.145% and 0.121% respectively) and adult (0.118% and 0.088% respectively) of *An. gambiae*. Similarly, the lethal concentration of *O. grattisimum* and *O. basilicum* to achieve 90% mortality was lower in larvae stage (0.363% and 0.246% respectively) compared to pupae (0.728% and 0.485% respectively) and adult (0.519% and 0.305% respectively) of *An. gambiae*. Larvicidal, pupicidal and adulticidal activity of the *Ocimum* extracts has been collaborated ted by the findings of other studies (1, 8, 19). The authors reported the effectiveness of *O. sellio* and *O. basilicum* essential oils on mosquitoes (19). Larvicidal, adulticidal, ovicidal, oviposition-deterrent and repellent activities towards three mosquito species were evaluated (1). Recently, Afolabi et al. (8) reported the adulticidal and repellent activity of different extracts of *O. caninum* and *O. gratissimum* against adult *An. gambiae*. Our present study, *O. basilicum* and *O. gratissimum* extracts significantly reduced the number of bites by the vector given a ranged of 72.25% to 81.75% protection.

Preliminary study on non-targeted organism, fingerlings of *C. garipienus* showed that *Ocimum* species at the tested concentrations did not cause lethal effects on the numbers of fingerlings introduced. No previous study on the effects of *O. basilicum* and *O. gratissimum* extracts on non-targeted organism, fingerlings of *C. garipienus* was found in literature. However, the addition of *O. americanum* to diet of *Sciaenops ocellapus* did not significantly affect the reproductive parameter and whole body composition after 7wk (20).

## Conclusion

Extracts of *O. grattisimum* and *O. basilicum* are promising in disease-vector mosquito’s management. These findings have demonstrated to be one of the alternative approach to manage mosquito vectors than the use of synthetic chemical insecticides that causes adverse effect on humans, environment and on non-target aquatic organisms. Further studies should be conducted to describe toxicological and histological effects of *Ocimum* species on non-target organisms, fingerlings of *C. garipienus*.
